# Disseminated Langerhans cell histiocytosis associated with acute myeloid leukaemia: complete remission with daunorubicin and cytarabine

**DOI:** 10.1007/s00277-012-1555-6

**Published:** 2012-08-19

**Authors:** Yu-Yan Hwang, Po Tsui, Rock Y. Y. Leung, Yok-Lam Kwong

**Affiliations:** 1 Department of Medicine, Professorial Block, Queen Mary Hospital, Pokfulam Road, Hong Kong, China; 2 Department of Pathology, Pamela Youde Eastern Hospital, Hong Kong, China; 3 Department of Pathology, Queen Mary Hospital, Hong Kong, China

**Fig. 1:**
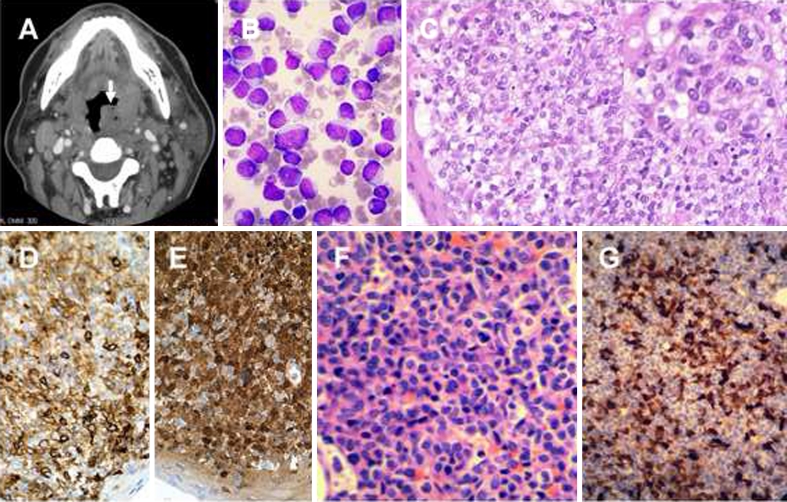
Concomitant disseminated Langerhans cell histiocytosis and acute myeloid leukaemia. **a** Computed tomography showing bilaterally enlarged tonsils, more prominent on the left side (*arrow*). **b** Marrow aspirate showing predominance of myeloblasts. **c** Tonsillar biopsy, showing infiltration by a population of atypical cells with indistinct cytoplasmic borders. High-power view (*insert*) showed atypical cells with nuclei containing linear grooves. **d** The tonsillar atypical cells were positive for CD1a (immunoperoxidase). **e** The tonsillar atypical cells were positive for S100. **f** Trephine biopsy of the marrow, showing admixture of myeloblasts and atypical cells with elongated and grooved nuclei. **g** Atypical cells in the marrow were positive for S100

Dear Editor,

A 68-year-old man presented with cervical lymphadenopathy and bilateral tonsillar enlargement and ulcerations. Computed tomography (CT) showed an infiltrative disease (Fig. 1a, arrow). A blood count showed haemoglobin, 13.1 g/dL; leucocytes, 41.9 × 10^9^/L (89 % blasts); and platelets, 48 × 10^9^/L. Bone marrow examination confirmed acute myeloid leukaemia (AML) without maturation (Fig. 1b). Cytogenetic analysis showed 46,XY,t(7;14)(p13;q32), the significance of which was unknown. *NPM1* and *FLT3* genes were wild type. Unexpectedly, a biopsy of the tonsillar mass showed infiltration by medium-sized atypical cells with indistinct cytoplasmic borders (Fig. 1c) and irregular and grooved nuclei (insert). These atypical cells were positive for CD1a (Fig. 1d) and S100 (Fig. 1e). Myeloblasts were not evident. Pathological features were consistent with Langerhans cell histiocytosis (LCH). Marrow trephine biopsy showed myeloblasts intermixed with numerous atypical cells (Fig. 1f), which were also S100 positive (Fig. 1g). The overall diagnosis was therefore consistent with concomitant disseminated LCH and AML. As leukaemia treatment was more urgent, a standard AML regimen of daunorubicin (60 mg/m^2^/day × 3 days) and cytarabine (100 mg/m^2^/day × 7 days) was administered. Interestingly, the tonsillar masses and cervical lymphadenopathy subsided, and a day-22 marrow examination showed complete remission of AML and LCH. A positron emission tomography/CT (PET/CT) also confirmed complete response of the LCH. Two consolidation courses of high-dose cytarabine have since been given, with the patient remaining in CR 6 months after diagnosis.

LCH in association with leukaemia occurs mainly in two clinical patterns: LCH preceded by acute lymphoblastic leukaemia (pathogenesis undefined) and LCH treated by etoposide/vinblastine followed by therapy-related AML [1]. Concomitant LCH and AML have very rarely been reported [2,3]. Two explanations for this extraordinary phenomenon have been proposed: LCH and AML deriving from the same neoplastic precursors or LCH being reactive to the AML [1–3]. In our patient, the LCH was infiltrative with marrow metastasis, supporting that it was neoplastic. In addition to its diagnostic interest, this case had therapeutic implication. The optimal treatment of disseminated LCH remains undefined. The use of vinblastine, etoposide, 2-chlorodeoxyadenosine, cyclophosphamide and cytarabine has been described [4]. Anthracyclines have apparently not been studied before. Our case showed fortuitously that a standard daunorubicin/cytarabine regimen was highly effective in inducing a remission for disseminated LCH, an observation that requires further validation.
